# Exploring impact of green finance and natural resources on eco-efficiency: case of China

**DOI:** 10.1038/s41598-024-70993-4

**Published:** 2024-08-30

**Authors:** Xu Fang, Osamah Ibrahim Khalaf, Wu Guanglei, Juan Felipe Espinosa Cristia, Salwa Almasabi

**Affiliations:** 1 School of Accounting, Hunan University of Finance and Economics, Changsha, China; 2 Department of Solar, Al-Nahrain Research Center for Renewable Energy, Al-Nahrain University, Jadriya, Baghdad Iraq; 3 Departamento de Ingeniería Comercial, Universidad Técnica Federico Santa María, 2390123 Valparaiso, Chile; 4 Department of Accounting, College of Business Administration, Princess Nourah Bint Abdulrahman University, P.O.Box 84428, 11671 Riyadh, Saudi Arabia

**Keywords:** Ecological efficiency, Green finance, FDI, Economic & social development, Ecology, Environmental social sciences, Energy science and technology

## Abstract

China ranks 160 out of 180 countries in terms of ecological efficiency, with an EPI score of 28.40 and a 10-year average change in score of 11.40. This article examines the impact of green finance and China’s natural resources on regional ecological efficiency using the Tobit regression model. The study uses the average yearly exchange rate to normalize dollar-related values and GDP to 2012 RMB using the price deflator. Variables used as explanatory tools include green financing, the availability of natural resources, and regional eco-efficiency. The results of the study imply that natural resources in eastern region of China are better managed as and have avoided the resource curse as compared to central and western regions. Resources temporarily support area economic and social growth. However, resource agglomeration locks many elements in the resource industry and degrades regional industrial development, generating environmental and social difficulties that may hinder regional economic progress. Given that Foreign Direct Investment (FDI) increases regional eco-efficiency after accounting for adjustment. The FDI positively correlated with ecological efficiency in the east zone, while central and western zones have negative correlations. The industrial development of the nation negatively impacts ecological efficiency in the East, Midwest, and West regions. Western results are distinctive, with ecological efficiency and regional economic growth frequently going hand in hand.

## Introduction

China’s economic growth has multiplied and strengthened. However, typical heavy development has degraded the biological environment. Regarding environmental performance, China ranks low compared to developed countries. The ranking of China in this respect, China ranks 160th out of 180 countries, with an EPI score of a mere 28.40 and a 10-year average change in score of 11.40, according to Ref.^1^. The main reason for the degradation of China’s ranking mainly relates to ever-increasing greenhouse gas emissions, leading to lower air quality. China stressed the importance of ecological civilization construction to boost economic growth and environmental carrying capacity. The old paradigm of forsaking the environment for economic growth is outdated. Changing the economic and social development paradigm and moving to a green economy is vital. Green development has spread across industries to implement the ecological civilization approach. The green economy has grown thanks to the government’s backing and leadership. Finance drives modern economic growth. Green finance is essential for a green economy. Green financing is vital for environmental protection and for avoiding future ecological disasters, significantly impacting the country’s overall economic progress and ecological efficiency^2^.

Literature suggests that the goal of eco-efficiency is to maximize economic output while minimizing ecological footprint and resource use. The focus of eco-efficiency studies has shifted from economic production to social services^3^. The focus of the prior research has not been on the individuals in the ecosystem. A second development is a shift from two-dimensional resource and environmental indicators to three-dimensional economic, social, and environmental indicators^4,5^. To date, assessments of human productivity have ignored fundamental aspects of human flourishing. The ecological economic system uses stage indicators, whereas GDP is used as a final output by researchers^6^. Although enhancing human well-being is a stated objective of sustainable development^7,8^. Chinese people’s standard of living has suffered due to the country’s rapid economic and social development and efforts to protect the environment^9^.

Green finance and environmental effectiveness have rarely been discussed in academic literature. Most are studied indirectly^10–14^, with environmental protection, economic and social development, and the modernization of industrial products all playing roles, with varying results. The scope of eco-efficiency has not expanded, the assessment index system has not been enhanced, and there is no consensus on the final product of the eco-economic system. This article examines the impact of green finance and China’s natural resources on regional ecological efficiency using the Tobit regression model, which stands used to assess the eco-efficiency of each province on the Chinese mainland from 2012 to 2021. This study’s research model examined regional variations in ecological efficiency, resource availability, and Green Finance in China. Results: Beijing’s, Shanghai’s, and Tianjin’s environmental performance are of the highest caliber. Qinghai, Gansu, Ningxia, Xinjiang, and Liaoning are below the national average of 0.55 in western China. Eco-efficiency is only fair in this case. According to data dynamics, most regions’ ecological effectiveness has increased, with the high east and low West frequently diverging. Ecological effectiveness and national green funds have a nonlinear connection. Green Finance is U-shaped and inversely connected with ecological efficiency in the central and western regions.

The novelty of the present study mainly relates to the fact that examined regional variations in ecological efficiency, resource availability, and Green Finance in China, while the previously this approach has been applied to entire country. Our study allows the policy makers to focus on the different polices for to account for Environmental effectiveness and industrial progress are at odds with one another. Given fact that environmental efficiency and regional economic growth go hand in hand. But the rate of tend to differ across three distinct geographical regions of China. This aspect has rarely been explored in previous studies.

## Literature review

According to Ref.^15^, eco-efficiency is a metric for assessing the performance of sustainable development programs that attempt to reduce the strain humanity places on the planet’s natural resources without compromising the quality of the products they generate. Eco-efficiency has emerged as a management response to waste issues associated with current production processes, making it one of the most analytical and quantitative strategies for organizations interested in practical ways to gain sustainable development^16^. Developing eco-indicators is essential for providing a more comprehensive understanding of the situation and facilitating decision-making procedures that will improve human well-being and safeguard ecological systems. Recent years have seen the creation of eco-efficiency indicators for products, a requirement for sustainable development. These metrics were created to address the many barriers to sustainability, including considering a product’s economic and environmental effects over its entire life cycle, discounting the impact of future actions, and combining multiple environmental harm indices into a single index^3^. For a business to succeed over the long term and to protect the environment, eco-efficiency indicators are crucial^17,18^. Businesses must now more than ever boost their social, financial, and ecological productivity.

It is impossible to have sustainable regions in an unsustainable globe. Thus, the indicators will only be helpful if they promote sustainable development worldwide. Therefore, it is essential to critically evaluate eco-indicators’ use from the perspective of sustainable development. However, as noted by Refs.^19,20^, academics and policymakers must continue discussing how they incorporate eco-efficiency concepts and indicators into their work, Eco-efficiency analysis is an effective communication tool incorporating a thorough, scientifically based approach to environmental impact assessment. One of the significant issues that can lead to errors in judgment mainly relates to incomplete or insufficient data. This issue may resolved by developing a comprehensive scale for systematically evaluating environmental, economic, and social indicators^19,20^.

Therefore, increasing eco-efficiency does not ensure a move toward sustainable growth. Even though it is advantageous, greater eco-efficiency alone will not guarantee the sustainability of industrialized societies because nuclear and renewable energy sources would have a significant adverse environmental impact, especially if adopted widely. Recycling and substitution cannot replenish the finite supply of nonrenewable minerals. The second rule of thermodynamics also states that industrial production systems have essential, unavoidable environmental repercussions. Ultimately, all the hard-earned improvements in eco-efficiency will soon be lost if population and consumption keep growing. Adjusting fundamental cultural attitudes that incorrectly associate material consumption and economic growth with well-being and happiness, as well as gains in eco-efficiency, is necessary^21^.

A significant obstacle in evaluating a region’s or nation’s environmental performance is the development of a metric for measuring eco-efficiency. The literature acts as a comprehensive manual in this regard^22^. Recent research has examined the nature and significance of eco-efficiency indices. In this respect, several techniques have been developed to quantify eco-efficiency, including energy analysis, index systems, material flow, ecological footprint, and data envelopment^23–25^.

For instance, a company’s material consumption would be the total of all the materials it purchases or receives^26^. Eco-friendly practices enable the creation of goods and services that use less material. The objective is to increase production while reducing input. As a result, reducing material consumption and increasing yield while using the same number of raw materials is possible. Energy is a vital input because it is used to maintain the process and provide heat and power. Energy sources such as biomass, nuclear power, hydropower, lignite, coal, oil, and gas may also be investigated^27^. Several valuable indicators for evaluating energy footprints include annual energy use, energy use per unit of production, and energy savings through efficiency programs. There are two ways to measure emissions: annually and per unit of output. Emissions from the air, water, and soil can all be calculated separately (waste)^28^. The computation includes values from energy, steam, and transportation production, resulting directly from these processes^29^. The GWP of additional greenhouse gases, such as O2, SF6, N2O, and CH4, must be considered to determine the total CO2-equivalent emissions^30^.

## Research methodology

The present study used OLS (Ordinary least-squares) based regression methodology. OLS models assume the study involves fitting a model that represents the relationship between one or more explanatory variables and a continuous or interval outcome variable. Reduce the sum of squared errors, the difference between the outcome variable’s actual and predicted values^31^. OLS models assume the analyst is fitting a model that represents the relationship between one or more explanatory variables and a continuous or interval outcome variable. Reduce the sum of squared errors, the difference between the outcome variable’s actual and predicted values. Another major reason for the choice of OLS relates to its simplicity. It requires no complicated math computations, making it easy to use. Efficiency is another benefit of this process^32^.

### Variable selection

The variables for the present research were selected after an in-depth review of the literature based on previous research on the given topic. The data availability for the variables was also considered, as the research was conducted solely in the Chinese context.

#### Green finance

Nations throughout the world are pushing the green economy. The green finance sector in China is expanding and making more significant investments in protecting the environment. Green development necessitates allocating social resources to ecological building, restoration, and pollution management. Green financing strengthens green industries, lowers environmental risks, and improves business environmental performance^33^. Green investments support the creation of natural reserves, the treatment of air, water, and sewage pollution, as well as the advancement of environmentally friendly industrial technologies^34^. The financial market absorbs money and channels it towards green, environmental protection sectors that support ecological building, pollution prevention, natural restoration, and environmental benefits, increasing ecological efficiency. China’s green financial products have increased, but they just recently began to be used^35^. The biggest and most advanced green financial tool is green credit. Since green credit is used, proxy factors for green economic development are more representative^36^.

#### Economic social development

This research uses the GDP per person indicator as a stand-in for economic progress. The model uses a base period of 2012 and an indicator for consumer Retail Price Index. This variable is correctly processed, i.e., presented in logarithms, to guarantee the unity of dimensions between the fundamental value of per capita Gross Domestic Product and the degree of eco-efficiency of the regions^37,38^. The co-integration link between the variables is protected when efforts are taken to remove heteroscedasticity from a dataset.

Given that the Heteroscedasticity refers to uneven dispersion. Heteroscedasticity in regression analysis relates to the residuals or error terms. To be more specific, heteroscedasticity refers to a continuous shift in the residuals’ distribution over the observed values. Ordinary least squares (OLS) regression assumes that all residuals are drawn from a population with a constant variance (homoscedasticity), making heteroscedasticity difficult. The residuals must be of constant variance to fulfill the regression assumptions and produce valid results.

#### Industrial development

Existing studies often utilize the share of tertiary industries in total industrial development as a proxy for measuring the pace of industrial growth. According to Ref.^8^, the primary, secondary, and tertiary energy usage patterns have consistently been low to high over time. Therefore, only at the tertiary level can accurate industrial development be displayed. Higher levels of living often lead to shifts in consumer preferences, which is good news for the tertiary sector. Both the expansion of the primary industry and the maturation of modern scientific knowledge have played essential roles. As the economy develops and modern science and technology advance, many parts of production move from the primary to the secondary and tertiary levels^38^. Because this research is primarily concerned with assessing regional eco-efficiency within China, previous research related to the topic has been used to fill in the gaps.

When we analyze the environmental impact of the industries, the secondary sector tends to have a more significant environmental impact when compared to the primary and tertiary^39^. In the end, the secondary sector’s contribution to regional GDP is used as a proxy for the research’s focus on the intensity of industrial development. If the ratio were higher, the impact would be even more detrimental to the environment and vice versa^40^.

#### Foreign direct investment

The probable impact of FDI on China’s regional ecological efficiency is demonstrated by FDI’s structural, income, and technological outcomes. FDI has affected ecological efficacy in different parts of China^41^. Facts show that FDI can positively and negatively affect the ecological efficiency of different locations in China. As a benchmark against the research of other specialists, this article uses the FDI-to-GDP ratio as it considered as a standard for measuring the share of investment in net production of goods and services and economy^42^.

### Source data

Many publications were consulted to compile the information presented here: the official website of the National Bureau of Statistics; the websites of provincial and municipal statistical offices; the “China Statistical Yearbook”; the “China Environmental Statistics Yearbook”; and the “China Banking Social Responsibility Report”. The average yearly exchange rate is used to normalize all dollar-related values. GDP is converted to 2012 RMB using the price deflator. The scale was constructed using previous research.

The details of these are presented in Table 1.

**Table 1:** Development of Scale for present research.

	Indicator	Sources	Unit of measurement	Authors
Indicators for input	Pollution	Water	Sewerage discharge in thousand KG	^43,44^
		Air	Sulfur-di-oxide in thousand KG	^45^
			Demand for commercially produced oxygen in thousand KG	^46^
			Cabon-di-oxide in thousand KG	^3,4,47^
			Emission of dust in thousand KG	^48^
		Solid waste	Production of garbage in thousand KG	^49^
	Consumption of energy and utilisation of natural resources	Water	The net amount of water utilized measured in (10,000 cubic meters)	^36^
		Energy	Million-Yuan GDP energy consumption	^30,50–54^
		Land	Construction land (km^2^)	^34^
Indicators for output	Economic and social development of the region		Regional gross domestic product in a million RMB	^38,40,55,56^

Table 1 contains the input indicators, which basically consists of different types of Pollutants i.e. Water, Air and solid waste. The water-based pollution is measured by Sewerage Discharge in thousand KG in the natural and manmade water ways^43,44^. The air-based pollution is measured by release of greenhouse gasses such as Sulfur-di-oxide, the demand for commercially produced Oxygen, release of Cabon-di-Oxide and increase in dust pollution as result of huma activities. These gasses not only increase the greenhouse effect but are also detrimental to human health. The inclusion of increase in dust particles in air relates to the ever-increasing deterioration of air quality in China especially in winter and spring seasons. Along with the well known effects of the greenhouse gasses the dust is one of the most prominent categories of naturally occurring aerosols, has a big effect on the radiative balance of Earth^48^. Economic and technological improvements, as well as governmental regulations, all impact solid waste management directly or indirectly. China is constantly improving its management system and investigating solid waste recycling, safe treatment, resource utilization, and source reduction. How industrial solid waste is created and processed will change due to the restructuring of the industrial structure. The quantity of household rubbish produced per GDP unit has decreased with urbanization. Reducing energy use, solid waste, and carbon emissions all occur together, necessitating financial investment and technological support^49^. Consumption of energy & utilisation of natural resources.

The second categories of input indicators consist of utilisation of natural resources such as water, land and energy to power the economic growth of China. According to the United Nations, China is one of 13 countries that lack adequate water supplies. Its huge expanse, diverse geography, and unequal distribution of water resources all contribute to this outcome, which is exacerbated by pollution. The net amount of water Utilized measured in (10,000 cubic meters) as held by Ref.^34^. Rapid land conversion for non-agricultural purposes has become a hallmark of Chinese urbanization. Land conversion for non-agricultural use was made more accessible by introducing township and rural enterprise development, infrastructure construction, and industrial and commercial development. The current land-use issues arose as China’s urbanization drive rapidly changed rural and urban areas. Many villages moved to cities during this period, and cities expanded quickly. Meanwhile, the demand for land is increasing as non-agricultural companies in small towns and rural areas expand. Nonetheless, certain obstacles may exacerbate land use issues and render interventions ineffective. Land utilization is measured by analyzing the construction land (km^2^).

The output indicator used is the economic and social development of different regions of China, and the Regional Gross Domestic Product measures are in a million RMB. One of the main distinctions of the present research is that all of these input indicators have previously been used, but to our knowledge, they have yet to be used in tandem.

#### Research model

Variables used as explanatory tools in this study include green financing (GrFin), the availability of the natural resource (NatRes), and regional eco-efficiency (EcoSocDev). Values are derived in this article by calculating EcoSocDev, IndDev, and FDI using the criterion mentioned in Table 1. To eliminate autocorrelation and address the issues related to multicollinearity, we resorted to taking the log values of all the variables from 2012 to 2021. We formulated the regression equation, presented below:

1 $$ {\text{ln EcoEffc }} = \alpha + \beta {\text{1 ln GrFin}}_{i} + \beta {\text{2 ln NatRes}}_{i} + \beta {\text{3 ln EcoSocDev}}_{i} + \beta {\text{4 ln IndDev}}_{i} + \beta {\text{5 ln}}FDI_{i} + \mu . $$

According to various figures, the average eco-efficiency is 0.872, which is on the lower end of the spectrum. There is substantial variation in the development of green finance, ranging from 400.021 to 2132.146, and notable differences are seen across different areas and years. Control variable statistics show that the average for regional economic and social development is 0.801, with the most outstanding value of 0.320 and the lowest value of 0.200. Between these two extremes, the average value is 0.801. Regarding foreign direct investment (FDI), the average value is 0.070, the maximum value is 0.200, and the lowest value is 0.009. The lowest value is 0.0000, while the most significant value is 50.323. The average value for the availability of natural resources is 4.023, with the highest value being 50.323. It is estimated that the median level of industrial development is 0.701, the highest level is 0.512, the lowest level is 0.160, and the highest level is approximately three times the lowest. Refer to Table 2 for the data that describes the situation.

**Table 2:** Descriptive statistics.

Description of variables	Variable symbol	μ	σ	Minimum	Maximum
Ecological efficiency	EcoEffc	0.872	12.211	0.3642	4.021
Green finance	GrFin	400.021	391.11	32.720	2132.146
Industrial development	IndDev	0.701	1.501	0.160	0.512
Availability of natural resources	NatRes	4.023	7.013	0.0000	50.323
Economic and social development	EcoSocDev	0.801	0.981	0.200	0.320
Foreign direct investment	FDI	0.070	0.301	0.009	0.201

### Ethics statement

The present research utilizes secondary data sourced from various sources such as the official website of the National Bureau of Statistics, the websites of provincial and municipal statistical offices, the “China Statistical Yearbook”, the “China Environmental Statistics Yearbook”, and the “China Banking Social Responsibility Report”, without involving any human or animal experimentation. The journal’s policy allows for the open access publication of the research findings. Aside from that, the research topic is general and does not pose any sensitivity for any group.

### Informed consent statement

Informed consent was obtained from all subjects involved in the study.

## Results and their implications

### Regional eco-efficiency

**Fig. 1:**
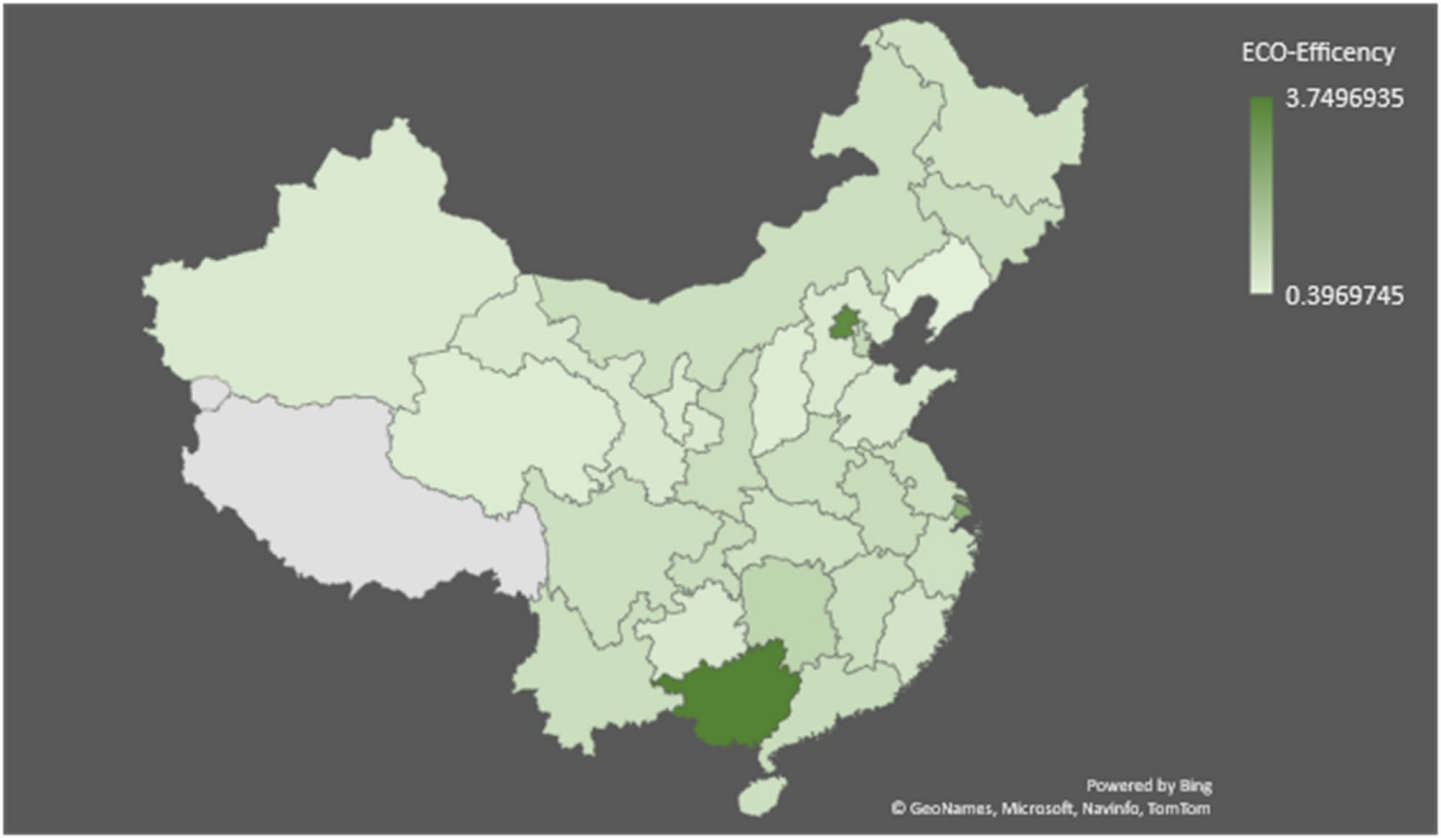
Map indicating the Eco-efficiency of Chinese cites. *Source* Present research. Figure was created via the 3-D Map chart function in MS-Excel for Microsoft 365, using the data collected for present research (https://www.microsoft.com/en-us/microsoft-365/excel).

**Fig. 2:**
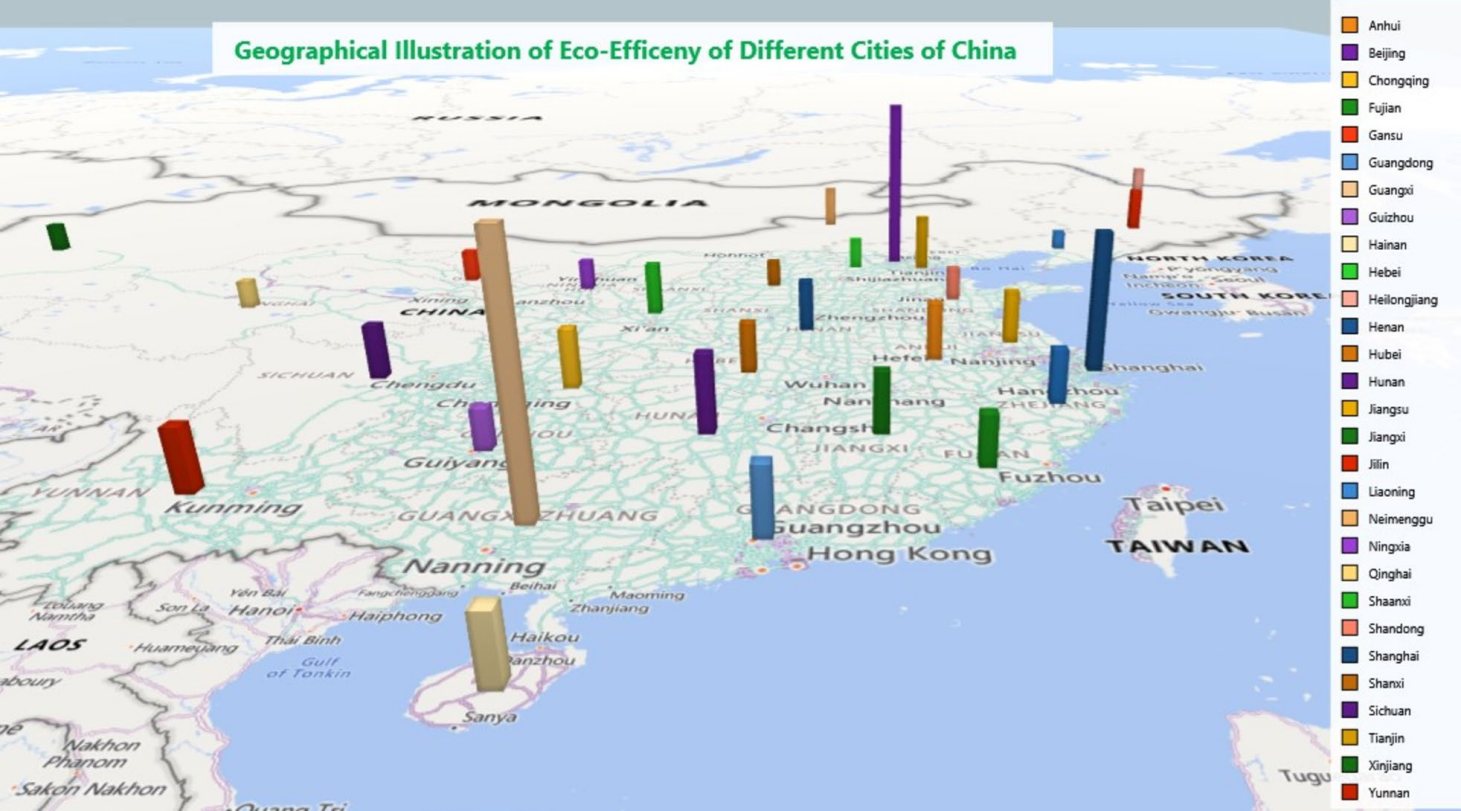
Geographical illustration of eco-efficiency of China. *Source* Present Research. Figure was created via the 3-D Map chart function in MS-Excel for Microsoft 365, using the data collected for present research (https://www.microsoft.com/en-us/microsoft-365/excel). The names of different regions and their corresponding colours are provided on the extreme right-hand side of figure. The height of indicates the values of eco efficiency scores of different Chinese cities.

**Fig. 3:**
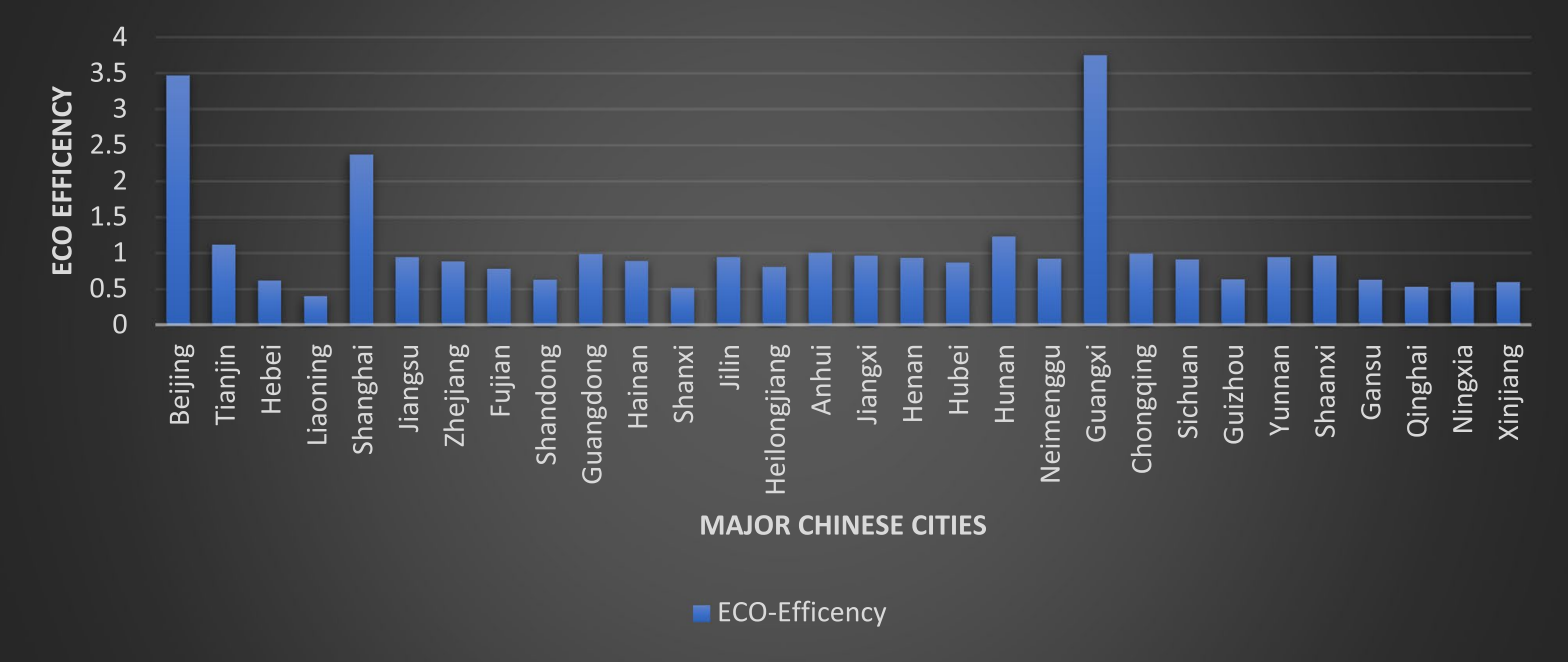
Average ECO-efficiency of Chinese cities from 2012 to 2021. *Source* Present Research.

Figures 1 and 2 represents the map and the Fig. 3 represent the graph illustrate China’s regional eco-efficiency from 2012 to 2021. Beijing and Shanghai have eco-efficiencies over 2.0, given that these regions are economically well-developed.

The government prefers sustainable, high-quality economic and social expansion in already-developed areas. After Beijing and Shanghai, areas with stricter environmental regulations and more stringent green development criteria are established. Ecological protection is relatively robust in developed economies, which helps persuade companies and governments to switch to greener forms of manufacturing and helps keep Beijing and Shanghai at the forefront of China’s ecological efficiency rankings.

Eco-efficiency indicators are calculated by dividing the value of a product or service by its environmental impact. Most indicators focus on the quantity of energy, materials, and use of natural resources such as land, water, and energy. Utilizing these resources results in the emissions of pollutants, such as greenhouse gases, wastewater, solid waste, and environmental changes, which might increase aerosols, such as dust, in the air.

The results of the study indicate that Qinghai, Gansu, Ningxia, Xinjiang, and Liaoning are below the national average of 0.55 in western China. The low ecological efficiency of the West can be traced back to the region’s undeveloped economy and social structure. Secondary industry in the West is largely self-sufficient.

The improved resource development results in less environmental impact. Investments from the West are harmful to the economy. In western China, pollution, emissions, energy consumption, and ecological inefficiency are all on the rise because the region prioritizes economic and social development over environmental conservation. Green practices in central China rapidly catch up to those in the east.

### Results Tobit model

Green financing promotes sustainable growth, green economic and social development, and regional ecological efficiency. Huge China. Regional economies differ. Green finance evolves. Regional variables will alter. The paper uses the Tobit regression model to examine how green finance and ample resources affect eco-efficiency across China from 2012 to 2021.Table 3 represents the regression results.

**Table 3:** Result of regression model.

Variable	Entire China	Eastern Region	Central Region	Western Region
Ln GrFin 2	0.031***	0.0231	0.3101***	0.1635*
	(3.99)	(0.51)	(5.41)	(1.80)
Ln GrFin	− 0.014***	− 0.0041	− 0.1014***	0.08013***
	(− 4.02)	(0.80)	(− 5.01)	(− 7.01)
LnNatRes	0.4001***	0.0036	0.4101***	0.0949***
	(4.01)	(− 0.75)	(4.06)	(3.00)
LnNatRes 2	0.0501***	0.0036	− 0.1301***	− 0.1510***
	(7.01)	(− 0.76)	(− 3.10)	(− 3.99)
LnEcoSocDev	0.007***	0.120***	− 0.0400**	− 0.0700***
	(3.31)	(2.15)	(2.05)	(3.99)
LNFDi	0.051***	0.1211***	− 0.1561***	− 0.2110***
	(7.012)	(4.00)	(4.10)	(3.96)
LnIndDev	− 0.130***	− 0.0511***	− 0.0916***	− 0.2334***
	(4.01)	(7.30)	(6.20)	(4.99)
Constant	− 2.2101	− 0.2141*	− 0.0700	− 0.3000***
	(0.35)	(1.81)	(− 0.31)	(− 7.15)

Green financing decreases ecological efficiency. Green finance’s first-order coefficient is negative, but its quadratic coefficient is positive and statistically significant. Efficiency does not increase linearly. “U-shaped feature low green financing development does not improve ecological efficiency. Assumption 1 is confirmed when green money encourages ecological efficiency at scale. This paper’s results fit the environmental Kuznets curve. Green financing and ecological efficiency have been found to be negatively correlated in central and western China. Green finance has substantial negative first-order and positive quadratic coefficients. Green finance and ecological efficiency form a “U” shape in two regions: “-shaped features, where lower green finance reduces ecological efficiency—increasing the volume of green financing after a critical mass boosts ecological efficiency. Green Finance improves ecological efficiency more in the middle than in the West. Green finance’s U-shaped association with the eastern region’s economy has negative first order and positive quadratic coefficients. First- and second-order variables are insignificant. Ecological efficiency requires more green financing.

The regression results demonstrate that the first term of resource availability is positive, and the second term is negative and significant at 5% and 1%. China’s resource abundance and ecological efficiency are negatively co-related. Accumulation within resource availability can boost regional green economic growth and environmental efficiency. Congestion from resource industry agglomeration limits ecological efficiency improvement, proving assumption 2. Eastern resources are scarce. Eastern regions need better management of natural resources. Eastern industrialization and institutions, mitigate the resource curse. Central and western resource availability tends to form an inverted U-shaped. Resources temporarily support area economic and social growth. However, resource agglomeration locks many elements in the resource industry and degrades regional industrial development, generating environmental and social difficulties that may hinder regional economic progress.

### Results of control variables

The elasticity value is 0.033, and the significance test demonstrates a significant positive link between FDI and the national sample’s ecological efficiency. The estimated results show that FDI has enhanced China’s ecological efficiency, but the degree of opening up of the middle and West of China negatively affects ecological efficiency. Regional ecological efficiency decreases with value. FDI has a more significant impact on the regions with inferior economic and social development. Consumption has harmed the environment, but Western consumption is much worse, showing that the central region’s economic and social development and utilization of foreign money are better than the West. In west and central regions, FDI may prioritize resource development and consumption, causing significant environmental damage. Thus, the estimated results are negative. Eastern FDI boosts ecological efficiency, but the elastic coefficient is small, indicating that foreign investment is better used in the East regarding eco-efficiency. FDI regional eco-efficiency improves because its benefits exceed its drawbacks.

According to three-region regression, industrial development and regional ecological efficiency are inversely correlated, indicating the importance of Elasticity. Industrial development negatively correlates with regional eco-efficiency in the eastern, central, and western regions, indicating that secondary industry has polluted and released harmful pollutants. Western industrialization has a more significant environmental impact with an elasticity of − 0.2334. Overreliance on the energy-intensive secondary sector makes Western regions inefficient. Manufacturing and technology in East China minimize pollutants and boost ecological efficiency. Industrial development centrally impacts eco-efficiency.

Ecological efficiency increases with economic expansion, and Elasticity is greater than 0. Eastern and national samples show positive changes, while central and western areas reverse with elasticity coefficients less than 0. Eastern economic growth prioritizes quality and industrial upgrading over western and central regions. Technology helps traditional businesses and the environment. Eastern regions have greater GDPs and environmental management resources. West and central regions overexploit resources and degrade the environment due to their slower economic growth and greater resource dependence than the East. It also suggests that central and western areas must adjust their financial growth strategy, vigorously promote clean and high-tech enterprises, and boost industrial development to address environmental issues caused by economic and social development.

### Discussions of results

There is a notable disparity in ecological efficiency between China’s eastern and western areas. The regional ecological efficiency of the Chinese country was evaluated by numerous academics, including Refs.^36,41^, using diverse methodologies. China’s regional ecological efficiency is representative at the regional level through several index selection and evaluation methodologies. This study diverges from previous research by identifying a nonlinear correlation between green financing and ecological efficiency on a national scale and in the central and western regions. The implementation of green money could enhance regional ecological efficiency. The participants’ nonlinear relationship can be attributed to the chosen research methodology. This article confirms the nonlinear relationship by studying a quadratic term variable. Representing the primary departure from the previous investigation. Nonlinear connection analysis assists in the formulation of policies. This study demonstrates the nonlinear relationship between ecological efficacy and resource availability across multiple scenarios^57^.

Only a few researchers have investigated the nonlinear relationship between the availability of resources and ecological effectiveness, with the majority focusing on a national scale. This shows that ecological efficiency and natural resource availability are not linear. Cities benefit from a natural resource abundance ranging from 8 to 15%. The nation cannot be maintained or continued in its current state^58^. Ultimately, this study advances regional ecological efficiency research and provides essential references for global ecological efficiency.

## Conclusion, policy implications and future research

### Conclusion

The study investigates regional variations in ecological efficiency, resource availability, and Green Finance in western China. Beijing, Shanghai, and Tianjin have the highest environmental performance, while Qinghai, Gansu, Ningxia, Xinjiang, and Liaoning are below the national average of 0.55. Eco-efficiency is only fair in these regions. China’s western region is divided into five regions, with most regions experiencing increased ecological effectiveness.

Green finance has a nonlinear connection with ecological efficiency in the central and western regions, with an inverted U-shaped association that is statistically significant. The study also shows that increased foreign direct investment (FDI) can improve regional eco-efficiency. The relationship between green financing and ecological efficiency is complex, with a U-shaped association in the center and western regions and a negative correlation in the central and western zones. The study also finds that ecological efficiency and regional economic growth go hand in hand in the West. The elasticity coefficient of GDP per capita-ecological efficiency is positive in the east, while it is negative in the center and West. The country’s industrial development negatively impacts ecological efficiency in the East, Midwest, and West regions.

In conclusion, ecological efficiency and regional economic growth in western China are closely linked. The eastern region shows a positive elasticity coefficient, while the middle and Western regions show a negative correlation.

### Policy implications

Excessive energy use in dwellings is the primary source of energy inefficiency. Thermal comfort, the quantity and energy efficiency of the equipment used, the local temperature, and the families’ financial situation all contribute to excessive consumption. The article’s results assist decision-makers in selecting policies that are most likely to be implemented in China’s economy and could receive funding and support from both the public and private sectors for growth and development. Promoting sustainable energy consumption through renewable energy sources and implementing legislation encouraging energy efficiency in conjunction with environmentally sound choices is critical.

Energy efficiency policies for a sustainable economy promote resource efficiency, sustainable growth, and reduced greenhouse gas emissions. Furthermore, the public sector has the potential to create new markets for energy-efficient products, services, and businesses. Optimizing efforts to improve energy efficiency in the housing sector is critical economically. More public-sector loan and subsidy programmes can help fund research, development, and investment in energy-efficient and renewable technologies. The notion is especially valid in China’s western areas, which could be more efficient due to an overreliance on energy-intensive secondary industries. As in East China, manufacturing and technical developments minimize pollution while increasing ecological efficiency. Industrial development has a direct impact on ecological efficiency.

### Future direction of the research

Exploring other energy intensity and economic complexity types would be prudent to determine whether the results are consistent. An alternative technique would be to investigate the impact of national characteristics such as demography, general climatic conditions, and population density, urbanization and industrialisation on the relationship between energy intensity and economic complexity. Finally, this topic can also be linked to other pressing global challenges, such as population growth, energy transition, global warming, and economic development.

## Data Availability

The data used in this study are available upon request from the corresponding author.
